# A novel Twinkle (*PEO1*) gene mutation in a Chinese family with adPEO

**Published:** 2008-11-03

**Authors:** Zhirong Liu, Yao Ding, Ailian Du, Baorong Zhang, Guohua Zhao, Meiping Ding

**Affiliations:** Department of Neurology, Second Affiliated Hospital, College of Medicine, Zhejiang University, Hangzhou, China

## Abstract

**Purpose:**

Autosomal dominant progressive external ophthalmoplegia (adPEO) is a genetically heterogeneous, adult-onset disease. Thus far, disease loci have been identified on four different nuclear genes. The purpose of this study is to identify the gene responsible for causing adPEO in a Chinese family.

**Methods:**

Clinical data and genomic DNA of a Chinese adPEO family were collected following informed consent. Gene scan by two-point linkage analysis was performed for four genes, and mutation screening was conducted in the Twinkle (*PEO1*) gene by direct sequencing.

**Results:**

A maximum two-point LOD score of 2.8 at θ=0.00 was obtained with marker D10S192 in close proximity to *PEO1*. A novel missense mutation (c.1423G>A, p.475A>T) was identified.

**Conclusions:**

This study widens the mutation spectrum of *PEO1* and is the first to report the *PEO1* mutation in the Chinese population.

## Introduction

Autosomal dominant progressive external ophthalmoplegia (adPEO, OMIM 157640) is an adult-onset disease with typical features including ptosis, external ophthalmoplegia, and slowly progressive skeletal muscle weakness. Some patients may also develop cardiomyopathy, cataracts, ataxia, peripheral neuropathy, major depression, and levodopa-responsive Parkinsonism. It was the first Mendelian disorder associated with multiple deletions of mitochondrial DNA (mtDNA) [1-3]. At the same time, adPEO is the most common genetically heterogeneous clinical entity belonging to the subgroup of human mitochondrial disorders caused by mutations in nuclear genes. Linkage analysis and the screening of candidate genes in different sets of families led to the discovery of pathogenic mutations in four different nuclear genes: the muscle-brain and heart-specific isoform of the adenine nucleotide translocator 1 (*ANT1*, on chromosome 4q), C10orf2 encoding a mitochondrial helicase (Twinkle, also named *PEO1*, on chromosome 10q24), the sole mtDNA polymerase gene (*POLG*, on chromosome 15q), and thymidine phosphorylase (*TP*, on chromosome 22q13.32) [4-8]. The estimated frequencies of mutations in adPEO have been reported to be 4%–10% for *ANT1*, 15%–35% for *PEO1*, and 45% for *POLG1*. *TP* is mainly associated with mitochondrial neurogastrointestinal encephalomyopathy (MNGIE) [3-6,9-13]

Herein, we describe a Chinese adPEO family with a novel mutation in *PEO1*.

## Methods

### Clinical evaluation and DNA specimens

**Figure 1:**
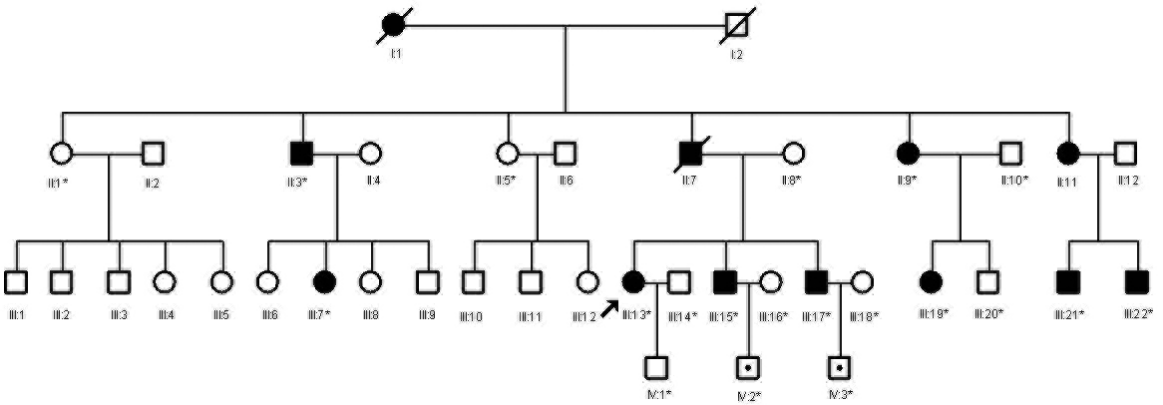
The pedigree of the Chinese family with adPEO. The index patient is indicated by an arrow. Family members who were sampled for linkage analysis and mutation screening are indicated by an asterisk. Affected family members are shown as darkened symbols. The mutation carriers who may be presymptomatic patients are indicated with dotted squares.

A four-generation adPEO family (Figure 1) was identified through the Department of Neurology of the Second Affiliated Hospital at the Zhejiang University College of Medicine in Hangzhou, China. Informed consent in accordance with the Zhejiang Institutional Review Board’s approved methods was obtained from all participants. Twenty individuals participated in the study, nine affected individuals and 11 unaffected individuals (Figure 1). All these family members were investigated according to their history of neurological examinations. A general clinical examination, which included tests of the blood lactate level, cranial magnetic resonance imaging (MRI), and muscle biopsies, was performed only in the proband. Fundus photographs were recorded by a TRC.50EX Retinal camera (Topcon Corp, Tokyo, Japan), and the electroretinograms were recorded on an LKC, UTAS-3000 (LKC Technologies Inc., Gaithersburg, MD). Twenty blood specimens were collected in EDTA, and leukocyte genomic DNA was extracted by standard methods. DNA (n=100) was extracted from ethnically matched, apparently healthy, anonymous adults (ranging in age from 18 to 65) composed of 42 females and 58 males.

### Muscle biopsy studies

Muscle biopsy of the biceps brachialis was performed on the proband under local anesthesia. Tissues were prepared for light and electron microscopic examinations (JEM-1230, JEOL, Tokyo, Japan). Frozen sections (10 mm thick) of biceps brachialis muscle were stained with hematoxylin-eosin (HE), modified Gomori’s trichrome stain (GT), adenosine triphosphatase (ATPase; pH 9.4, 4.3, and 4.6), NADH-tetrazolium reductase, and succinate dehydrogenase (SDH).Total DNA was extracted by standard methods from the muscle biopsy of the proband.

### Genotyping and linkage analysis

The same group of 20 family members was genotyped with 11 microsatellite markers surrounding the locus of *ANT1*, *PEO1*, *POLG1*, and *TP* [4-8]. Alleles were analyzed by GENESCAN Analysis version 3.0 and GENOTYPER version 2.1 software (Perkin-Elmer Applied Biosystems, Foster City, CA). Two-point LOD scores were calculated by the MLINK program of the LINKAGE package (version 5.1). The disease was specified to be an autosomal chromosome dominant trait with penetrance of 0.9 in affected individuals. The allele and recombination frequencies were assumed to be equal in males and females. We assumed gene frequencies of 0.0001, and no sex difference in recombination.

### Direct sequencing and mutation analysis

A strong candidate gene, *PEO1*, was polymerase chain reaction (PCR) amplified using the primers, Tw-Ex1–4 and Tw-In1R, as published previously [6]. Direct sequencing of the amplified fragments was performed on an ABI Prism 3130 Genetic Analyzer (Applied Biosystems, Foster City, CA). Sequencing results were assembled and analyzed using the SeqMan II program of the Lasergene package (DNA STAR Inc., Madison, WI). For all samples containing an abnormal *PEO1* amplicon, new PCR products were re-amplified from genomic DNA using the same protocols. Cosegregation analysis was performed.

### Long-range polymerase chain reaction for mtDNA deletions

Long-range PCR (TaKaRa LA Taq; Takara Bio Inc, Shiga, Japan) for mtDNA deletions was performed as previously described using a forward primer, nt 8285–8314, and a reverse primer, nt 15574–15600, with an annealing temperature of 63.0 °C [13].

## Results

### Clinical evaluation

**Figure 2:**
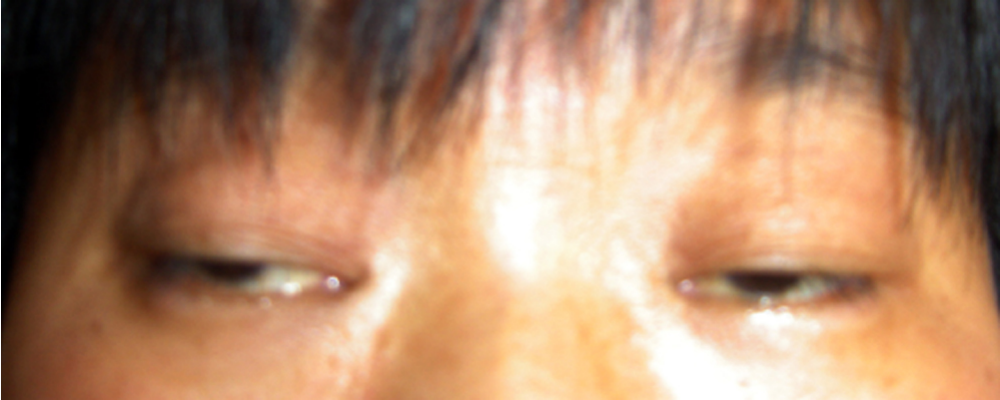
Clinical photograph of the proband’s extraocular muscles. Photograph of the proband show bilateral progressive ptosis and external ophthalmoplegia affecting all external ocular muscles.

The pedigree is shown in Figure 1. The proband (III:13 in Figure 1) is a 44-year-old woman who had bilateral progressive ptosis for eight years and had a three year history of diplopia. Neurological examination revealed an incomplete external ophthalmoplegia affecting all external ocular muscles and bilateral ptosis (Figure 2). There was no limb weakness or other abnormal neurological or psychiatric features. A general clinical examination did not reveal any abnormalities. At rest and during moderate exercise, the subject’s blood lactate levels were all normal. Serum creatine kinase and electromyogram of the limb muscle were normal as was the MRI of the brain. The fundus photographs and the electroretinograms were also normal.

The proband’s 60-year-old aunt (II:9) has bilateral ptosis, which first developed in her early 30s, and external ophthalmoplegia. She has a history of hypertension and lacunar infarction in the basal ganglia. At the age of 57, she developed rigidity. However, she shows no resting tremor or slurred speech. Additionally, she was found to be unresponsive to levodopa and diagnosed with Parkinson syndrome.

The proband’s 68-year-old uncle (II:3) complained of bilateral external ophthalmoplegia and ptosis at the age of 35. Both of her brothers (III:15 and III:17) were unaware of any clinical abnormalities in themselves, but subsequent examinations revealed mild ptosis and opthalmoparesis in each. Neurological examinations of individuals IV:2 and IV:3 revealed no symptoms. Other family members were clinically examined and are shown in Table 1. None of the affected family members showed limb weakness or other neurological or psychiatric abnormalities.

**Table 1:** Clinical information on the family with adPEO.

**Individual**	**Clinical feature**	**Age/ onset age**	**Neuroimage**	***PEO1*** **mutation**
II: 1	Normal	72/-	NP	NO
II: 3	severe restriction of horizontal and vertical eye movements	68/35	NP	YES
II: 5	Normal	65/-	NP	NO
II: 8	Normal	63/-	NP	NO
II: 9	severe restriction of horizontal and vertical eye movements, Parkinson syndrome	60/30	lacunar infarction in the basal ganglia	YES
II:10	Normal	60/-	NP	NO
III: 7	diplopia and severe restriction of horizontal and vertical eye movements	45/33	NP	YES
III:13 (proband)	Ptosis, severe restriction of horizontal and vertical eye movements	44/35	Normal	YES
III: 14	Normal	44/-	NP	NO
III:15	Ptosis, mild restriction of horizontal eye movements	36/32	NP	YES
III:16	Normal	33/-	NP	NO
III: 17	Ptosis, mild restriction of horizontal eye movements	34/32	NP	YES
III:18	Normal	32/-	NP	NO
III:19	Ptosis, diplopia and mild restriction of horizontal eye movements	30/30	NP	YES
III:20	Normal	28/-	NP	NO
III:21	Ptosis, diplopia and mild restriction of horizontal eye movements	33/30	NP	YES
III:22	Ptosis, diplopia and mild restriction of horizontal eye movements	31/30	NP	YES
IV:1	Normal	17/-	NP	NO
IV:2	Normal	6/-	NP	YES
IV:3	Normal	4/-	NP	YES

This table describes the clinical information on affected and unaffected members in family members. “NP” stands for ” not performed.”

**Figure 3:**
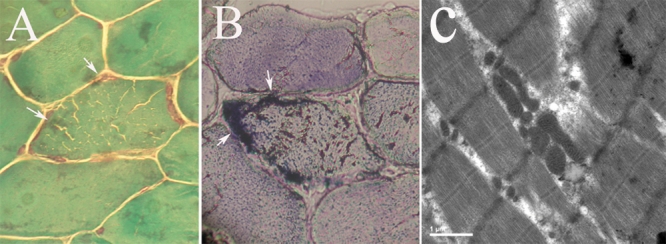
Light microscopic examination and electron microscopic examination of a 10 μm skeletal muscle transverse section. Gomori’s trichrome stain showed possible red ragged fiber (RRF; **A**). Succinate dehydrogenase (SDH) staining showed enhanced staining of the fiber edges (see arrows in **B**). Electron microscopic examination showed mitochondrial proliferation and enlarged mitochondria in a few muscle fibers, there were no crystalloid inclusions (**C**).

Muscle biopsies of the proband were analyzed. Gomori’s modified trichrome stain showed scattered muscle fibers and possible ragged red fibers (RRFs; magnification 40X; Figure 3A). SDH staining showed enhanced staining of the fiber edges (Figure 3B). NADH stain showed normal type I fibers. ATPase reaction showed the normal ratio of fiber type I and II. HE staining revealed mild variability of fiber size without necrosis (data not shown). Electron microscopic examination showed mitochondrial proliferation and enlarged mitochondria in a few muscle fibers but without crystalloid inclusions (Figure 3C).

### Linkage analysis

Candidate loci related to adPEO were initially screened with 11 markers. Two-point maximum likelihood data for markers of this region is summarized in Table 2. Two-point LOD scores were generated with markers D10S192, D10S597, and D10S185. The highest observed LOD score was 2.80 (θ=0.00) with marker D10S192. The relation of the other genes (*ANT1*, *TP*, and *POLG*) to adPEO was not supported by the genetic analysis, but significant linkage was found with markers of the *PEO1* locus in the chromosome 10q23.3–24.3 region.

**Table 2:** Linkage results with markers from the genes *ANT1*,*PEO1*, *POLG1*, and *TP*.

**Candidate** **gene**	**Markers**	**LOD SCORES AT θ=**					
		**0**	**0.1**	**0.2**	**0.3**	**0.4**	**0.5**
*ANT1*	D4s1535	−0.39	−0.3	−0.2	−0.09	−0.02	0
	D4S426	−0.39	−0.31	−0.21	−0.1	−0.03	0
*PEO1*	D10S185	1.58	1.26	0.94	0.6	0.27	0
	D10S192	2.8	2.27	1.68	1.03	0.39	0
	D10S597	1.53	1.23	0.91	0.59	0.27	0
*POLG1*	D15S205	−0.6	−0.28	−0.12	−0.05	−0.01	0
	D15S127	−4.58	−0.5	−0.21	−0.08	−0.02	0
	D15S130	−0.26	−0.16	−0.09	−0.04	−0.01	0
*TP*	D22S423	−0.26	−0.17	−0.1	−0.04	−0.01	0
	D22S274	−4.25	−0.44	−0.18	−0.06	−0.01	0

Two-point LOD scores with markers from the genes *ANT1*, *PEO1*, *POLG1*, and *TP* are shown. The maximum two-point LOD score (2.8) was achieved at D10S192 at θ=0.

### Mutation analysis

**Figure 4:**
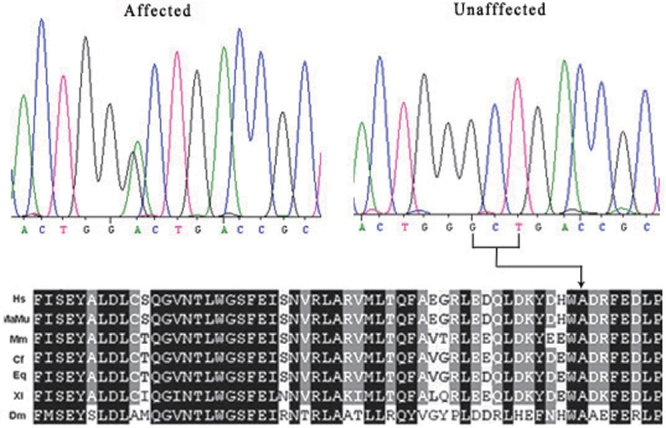
DNA sequence chromatograms of *PEO1* for unaffected and affected members in the family. A single transition was observed at position 1423 (G>A) of *PEO1*, causing a substitution of Ala to Thr at codon 475 (A475T). A table of phylogenetic conservation is also shown. Hs, *Homo sapiens*; MaMu, *Macaca Mutallata*; Mm, *Mus musculus*; Cf, *Canis familiaris*; Eq, *Equus Caballus*; Xl, *Xenopus laevis*, Dm, *Drosophila melanogaster*.

Through sequencing of *PEO1*, we found a base change (c.1423G>A, p.A475T; Figure 4) at position 1423 of the *PEO1* cDNA. This mutation has not been reported previously and cosegregated with all affected members in this Chinese family but was not detected in the 100 unrelated normal controls or in the unaffected pedigree members (Figure 4).

### mtDNA deletions analysis

**Figure 5:**
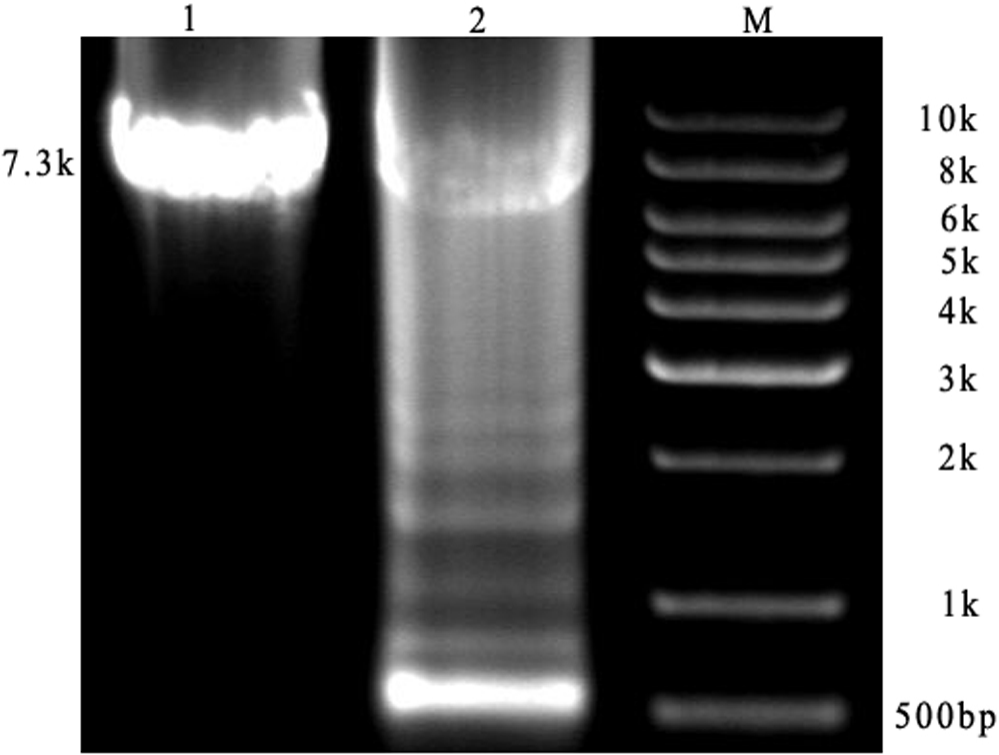
Long-range PCR for mtDNA deletion. Lane 1 shows the muscle DNA of a normal control while lane 2 shows the muscle DNA of the proband. M is a molecular weight marker. The multiple mtDNA deletions in the proband can be observed, but not in the normal control.

The results of the long-range PCR analysis are shown in Figure 5. The amplification of the 7.3 kb mtDNA fragment showed multiple deletions in the muscle DNA samples taken from the proband, which were absent in the normal control.

## Discussion

In this study, the highest LOD score of 2.80 was obtained at marker D10S192, strongly suggesting this adPEO family mapped to the locus on 10q24. This study also identified a novel missense mutation in *PEO1* (c.1423G>A, p.A475T). The pathogenicity of the *PEO1* mutation is based on the following criteria: (1) it results in an amino acid exchange from the amino acid alanine to the amino acid threonine; (2) the mutation harbors conserved residues, suggesting that this residue is functionally significant to the protein; and (3) we show that all living affected members of this family harbored the mutation while the mutation was not found in 100 unrelated, healthy individuals, a result highly suggesting that the c1423 G>A mutation segregates with the disease. The p.A475T mutation identified in this study widens the mutation spectrum of *PEO1*. Identification of the p.A475T missense mutation also confirms the causative role of the *PEO1* mutation. Interestingly, the p.A475P mutation has already been reported in adPEO patients of different ethnic backgrounds [6,14] so it may be assumed that A475 is a mutation hotspot. Variations in phenotypes are also seen in other adPEO families affected by the p.A475P mutation. From a clinical point of view, p.A475P mutations in *PEO1* are heterozygotes with dominant phenotypes. The patients may have a negative family history of adPEO, but they had proximal muscle weakness, ataxia, neuropathy, depression or avoidant personality traits, pes cavus, and tremors in addition to progressive external ophthalmoplegia.

PEO is characterized by multiple mitochondrial DNA deletions in the skeletal muscle. Both autosomal dominant and autosomal recessive inheritance can occur with autosomal recessive mutations of *PEO1* often associated with more severe symptoms such as early onset encephalopathy with mtDNA depletion [3,9-11,13]. Twenty-one different mutations have been identified in *PEO1*of which 20 are missense mutations. Mutations in *PEO1* may be of variable severity, being associated with clinical presentations ranging from late-onset “pure” PEO to PEO complicated by proximal limb and facial muscle weakness, dysphagia and dysphonia, mild ataxia, and peripheral neuropathy. According to previous research [6,9,10,12-19], mutations of *PEO1* are present in approximately 35% of familial PEO with multiple deletions of mtDNA. Therefore, *PEO1* mutations appear to be a common cause of family adPEO. No correlation has been found between specific mutations and the severity of clinical features [9-17].

In this family, the affected individuals with the A475T substitution had a relatively mild clinical phenotype (mainly represented by PEO), and the muscle biopsy exhibited fairly subtle changes without clear signs of multi-systemic involvement. Patient II:9 was unresponsive to levodopa and diagnosed with Parkinson syndrome. This is different from familial Parkinsonism because this patient’s syndrome was due to a mutation in *PEO1* [20]. Consideration of all of these reported mutations in *PEO1* might be important in deciding which gene to investigate in other families presenting with adPEO. In the proband, sensitive long-range PCR analysis revealed multiple mtDNA deletions in the muscle samples, but multiple mtDNA depletions were not shown in a normal control, indicating a severely compromised *PEO1* function in the proband. MtDNA deletions are typical for the muscle of adults with dominant *PEO1* mutations and are thought to accumulate by age, but there is not an obvious correlation between mtDNA deletion load and clinical presentation [6,13].

Normal human mitochondrial function involves a highly complex interaction between the nuclear and mitochondrial genomes. Hence, mutations in both mitochondrial and nuclear encoded genes can cause mitochondrial dysfunction and disease. *PEO1*, which is responsible for adPEO, encodes a protein with homology to the T7 gene 4 protein (gp4) [6]. *PEO1* is important for mtDNA maintenance, and its mutations are associated with progressive external ophthalmoplegia with multiple mtDNA deletions [21,22]. Interestingly, many mutations are clustered in a region of the Twinkle protein that is probably involved in subunit interactions. In addition, the A475 residue is located at the beginning of a very small helix in the helicase domain and is in contact with the R374 present in the linker region of the neighboring monomer. The A475 residue is conservative in terms of van der Waals interactions, but a proline residue may induce a dramatic change in the nature of the protein–protein interactions between the two monomers [21]. These findings suggest that the A475T mutation is a disease-causing change in *PEO1*.

In summary, our results reveal not only that a mutation in *PEO1* is associated with a Chinese adPEO family but also that the mutation spectrum of *PEO1* is expanded. This is the first report of the *PEO1* mutation in the Chinese population.
